# Examining the blood amino acid status in pretherapeutic patients with  hyperphenylalaninemia

**DOI:** 10.1002/jcla.23106

**Published:** 2019-11-24

**Authors:** Lili Liang, Jun Ye, Lianshu Han, Wenjuan Qiu, Huiwen Zhang, Yongguo Yu, Tianwen Zhu, Feng Xu, Xia Zhan, Peizhong Bao, Wenjun Ji, Xuefan Gu

**Affiliations:** ^1^ Department of Pediatric Endocrinology/Genetics Shanghai Institute for Pediatric Research Xin Hua Hospital Affiliated to Shanghai Jiao Tong University School of Medicine Shanghai China; ^2^ Department of Neonatal Medicine Xin Hua Hospital Affiliated to Shanghai Jiao Tong University School of Medicine Shanghai China

**Keywords:** amino acids, hyperphenylalaninemia, newborn screening, phenylalanine hydroxylase deficiency, phenylketonuria

## Abstract

**Background:**

Hyperphenylalaninemia is the most common genetic metabolic disease. Early treatment prevents brain injury effectively. The present study aimed to detect the exact amino acid status of patients with hyperphenylalaninemia before treatment.

**Methods:**

Data of 116 newborn patients from our Newborn Screening Center and 161 older patients from our clinic before treatment were collected. The content of 17 amino acids in their blood was determined by tandem mass spectrometry and compared with normal controls. Relationship between phenylalanine and other amino acids in patients was analyzed using the smoothing curve fitting and threshold effect analysis.

**Results:**

Most amino acids in the blood of patients were within the normal range; however, they were different significantly from those of the normal children. Newborn patients showed higher phenylalanine (346.30 vs 45.90 µmol/L), valine (121.50 vs 110.30 µmol/L), citrulline, ornithine and lower tyrosine (52.97 vs 66.12 µmol/L), threonine (68.68 vs 78.21 µmol/L), glutamine levels than observed in normal newborns. Older patients showed significantly higher phenylalanine (844.00 vs 51.82 µmol/L), valine (117.60 vs 110.90 µmol/L), histidine, serine and lower tyrosine (55.97 vs 67.31 µmol/L), threonine (35.94 vs 51.89 µmol/L), alanine, asparagine, glutamic acid, methionine, arginine, glycine, ornithine, glutamine content than found in matched normal children. Tyrosine, valine, ornithine, and threonine in newborn patients and tyrosine, glycine, glutamine, and threonine in older patients had a nonlinear correlation with phenylalanine levels with obvious threshold effect and clear inflection points.

**Conclusion:**

Significant difference was observed in the amino acid status between pretherapeutic hyperphenylalaninemia patients and normal children. Some amino acids showed notable threshold effect with phenylalanine level in a nonlinear pattern.

**Abbreviations**
Ala: alanine
Arg: arginine
Asp: aspartic acid
BH4: tetrahydrobiopterin
BMI: body mass index
CI: confidence interval
Cit: citrulline
Gln: glutamine
Glu: glutamic acid
Gly: glycine
His: histidine
HPA: hyperphenylalaninemia
Ile: isoleucine
Leu: leucine
LNAA: large neutral amino acid
Met: methionine
MS/MS: tandem mass spectrometry
NBS: newborn screening
Orn: ornithine
PAH: phenylalanine hydroxylase
PAHD: phenylalanine hydroxylase deficiency
Phe: phenylalanine
PKU: phenylketonuria
Ser: serine
Thr: threonine
Trp: tryptophan
Tyr: tyrosine
Val: valine

## 1 INTRODUCTION

Amino acid is the basic nutrient of the human body and the structural unit of proteins. Both proteinogenic amino acids and nonproteinogenic amino acids play an important role in biosynthesis, energy supply, and neurotransmission. For example, tyrosine (Tyr) and its precursor phenylalanine (Phe) are precursors of the catecholamine neurotransmitters dopamine, epinephrine, and norepinephrine and various trace amines. Glycine (Gly) is a precursor of porphyrins such as heme. Glutamine (Gln) is the precursor of glutamic acid (Glu), and Glu is the main excitatory neurotransmitter in the human brain.^1^ Arginine (Arg) is a precursor of nitric oxide.^2^ Aspartic acid (Asp), serine (Ser), histidine (His), Gly, and Gln are precursors of nucleotides.^3^ Tryptophan (Trp) is a precursor of the neurotransmitter serotonin.^4^ Nonproteinogenic amino acids in humans, including gamma‐amino‐butyric acid (GABA), citrulline (Cit), and ornithine (Orn), are key metabolic intermediates, such as in the biosynthesis of the neurotransmitter and so on. For example, Orn and S‐adenosylmethionine are precursors of polyamines.^5^

In the clinical setting, multiple amino acids can be determined in dried blood spots by tandem mass spectrometry (MS/MS). Changes in amino acid profile may be found in patients with various inborn errors of metabolism. Hyperphenylalaninemia (HPA) is the first disorder to benefit from newborn screening (NBS). HPA is a metabolic condition characterized by high serum Phe levels, caused mostly by phenylalanine hydroxylase (PAH) deficiency and seldom by tetrahydrobiopterin (BH4) deficiency.^6^ A recent recognition of the DNAJC12 deficiency, which is the co‐chaperone of PAH, expanded the clinical and metabolic spectrum of HPA.^7^ The term HPA should encompass all of the above conditions. PAH deficiency, also called phenylketonuria (PKU, OMIM no. 261600) and accounting for 90% of HPA cases, is the most frequent inherited amino acid metabolic disease with the incidence of approximately 1:11 000 in China, leading to the accumulation of Phe and hence brain injury. A Phe‐restricted diet is the primary effective therapy to prevent neurological damage in patients with PKU.^8^, ^9^ HPA is marked by a serum Phe level of >120 µmol/L and serum Phe/Tyr level of >2.^10^ HPA was classified according to the following cut‐off values of Phe concentrations at the time of testing: mild (non‐PKU) HPA (blood Phe: 120‐600 µmol/L), mild PKU (blood Phe: 600‐1200 µmol/L), and classical PKU (blood Phe: >1200 µmol/L).^11^, ^12^ In the 1960s, PKU became the first disease identified through the universal NBS program. Today, NBS in Shanghai is performed by MS/MS, which detects multiple amino acids besides Phe.

Although strict dietary adherence prevents profound neurologic injury, subtle cognitive deficits remain persistent in some patients who were treated early and received continuous treatment.^8^ Myelin abnormality, which is marked by impairment in the white matter integrity and deficiencies in the dopamine levels, may also involve abnormal amino acid metabolism.^13^, ^14^ However, this finding cannot fully explain the general disturbance of neuronal function and other underlying convergent mechanisms.

With regard to the amino acid metabolism in PKU, most previous studies focused on the “large neutral amino acid (LNAA) hypothesis.” LNAAs include Phe, valine (Val), Gln, threonine (Thr), Tyr, His, methionine (Met), leucine (Leu), Trp, and isoleucine (Ile). They are transported to the brain by the same amino acid transporter in the blood–brain barrier. In PKU, elevated plasma Phe levels impair the transport of other LNAAs, resulting in cerebral depletion of LNAA and hence decreased biosynthesis of protein and impaired brain function.^8^, ^15^ Increased cerebral Phe competes with Gly and Glu for their receptors, changing the levels of receptor expression and thus influencing the synaptic function.^16^ A previous study reported that the glutamatergic synaptic transmission in the brain is impaired in patients with PKU.^17^ PKU patients have reduced myelin formation and dopamine levels.^18^ However, only a few studies evaluated the serum amino acid status of PKU patients before treatment. The present study aimed to determine whether the status of other amino acids, except Phe, in patients with  HPA is disturbed prior to treatment. To clarify the amino acid status of HPA patients before treatment, we reviewed the amino acid spectrum of 277 patients in the past 15 years. We compared the patients’ amino acid spectrum with that of normal children and got some interesting results. Then, we analyzed the correlation between amino acid levels and Phe at different concentrations. As a result, significant results were also exposed.

## 2 MATERIALS AND METHODS

### 2.1 Study population

Since the normal reference ranges of amino acids differ with age, we showed reference range of amino acids level for newborns and older children separately. The study participants were divided into four groups. We reviewed the NBS program in our screening center from 2003 to 2018 and collected the data of 116 HPA newborns; their blood samples were usually collected on the 4th day after birth after fully feeding for 3 days (group 1). A total of 150 healthy newborn controls were selected randomly from the NBS database in the same birth period with newborns in group 1 (group 2). Another 161  HPA patients, aged 1 month to 5 years old, who were suspected to have HPA by other hospital, transferred to our clinic at several months of age mostly, and finally confirmed with HPA in our center from 2003 to 2018 were selected and comprised group 3. All the patients seemed stature symmetrical with normal weight and height when they visited our clinic. All the registered body mass index (BMI) values were within normal range according the WHO standards. Those with other accompanying chronic diseases were excluded. A total of 200 blood spot samples from age‐matched healthy children were used as control (group 4). Blood samples were collected while the patient was in a fasting state, and the samples detected within 48 hours after blood collection. The patients’ screening results were analyzed and compared with their respective controls. Furthermore, the correlation between the differential amino acid levels and Phe concentrations was also analyzed in different age groups.

### 2.2 Analytical methods

The amino acids in dried blood pots on filter paper were determined by MS/MS (Applied Biosystems, API 2000). All the chemicals used in MS/MS (methanol, and acetonitrile) were liquid chromatographic/mass purity grade and were purchased from Fisher Scientific. The preparation and detection methods were described in previous studies.^19^, ^20^ In brief, dried blood spots were extracted using methanol, which contained amino acids as internal standards. After derivatization with n‐butyl alcohol hydrochloric acid, the samples were tested using MS/MS. A total of 17 amino acids were determined, including Phe, Tyr, alanine (Ala), Asp, Glu, Met, Val, Arg, Gly, Gln, His, Ser, Thr, Leu, Trp, Cit, and Orn.

### 2.3 Statistical analysis

The results were expressed as arithmetic mean ± standard error of the mean (mean ± SEM). *T*‐test was used to study the differences in the concentrations of amino acids. Statistical analyses were performed using the SPSS version 22 statistical software (SPSS Inc). The correlation between the differential amino acid levels and Phe concentrations was analyzed using the EmpowerStats software. Using smoothing curve fitting, we determined whether a specific amino acid and Phe have linear or nonlinear correlation. We used Pearson's regression to determine the linear correlation between variables and threshold effect analysis for curve correlation. A *P* value of <.05 was considered significant.

### 2.4 Ethics statement

The study protocol was approved by the Ethics Committee of Xin Hua Hospital Affiliated to Shanghai Jiao Tong University School of Medicine (XHEC‐D‐2014‐141). Parents were properly informed about the study and signed a written consent.

## 3 RESULTS

### 3.1 Baseline characteristics of patients and controls

The characteristics and Phe concentrations of the four groups are summarized in Table 1. Group 1 included 116  HPA newborns, with the mean age of 4 days. The average Phe concentration in group 1 was 346.3 µmol/L, with a maximum concentration of 947.1 µmol/L and minimum concentration of 126.1 µmol/L. Most of the newborns had a pretherapeutic Phe concentration of 120‐600 µmol/L, which accounts for 90% of the group, while only 11 of them had a Phe concentration of >600 µmol/L.

**Table 1:** Characteristics and blood phenylalanine concentration of patients and controls

	Group 1 (n = 116)	Group 2 (n = 150)	Group 3 (n = 161)	Group 4 (n = 200)
Gender (M/F)	116（64/52）	150（83/67）	161（95/66）	200（114/86）
Age (mean ± SD, days)	4.78 ± 0.28	4.27 ± 0.05	103.40 ± 25.34	91.76 ± 20.86
Phe concentration (µmol/L)				
Mean ± SEM	346.30 ± 16.45	45.9 ± 0.84	844.00 ± 50.72	51.82 ± 1.13
<120	0	150/100%	0	150/100%
120‐600	105/90%	0	74/46%	0
600‐1200	11/10%	0	48/30%	0
>1200	0	0	39/24%	0

Abbreviation: SEM, standard error of the mean

Group 2 included 150 normal newborns, who were selected randomly from the NS database in the same birth period as newborns in group 1. Newborns (4 days old) in group 2 were age‐ and sex‐matched with those in group 1. Group 3 included 161 HPA older children with a mean age of 103.40 days. Despite the large age span in group 3, from 1 month to 5 years, 96% of them (154/161) came to our clinic at the age of 1 year. The average Phe concentration was 844 µmol/L, with a maximum concentration of 2997 µmol/L and a minimum concentration of 121.6 µmol/L. Among them, 74 had a Phe concentration of 120‐600 µmol/L, which accounted for 46% of the patients in the group; 48 had a Phe concentration of 600‐1200 µmol/L; and 39 had a Phe concentration of >1200 µmol/L, which accounted for 24% of the patients in the group. Group 4 included 200 healthy children. They were assigned as the control of group 3, with normal Phe, and were age‐ and sex‐matched with group 3.

### 3.2 Pretherapeutic amino acid status in  HPA patients

**Figure 1:**
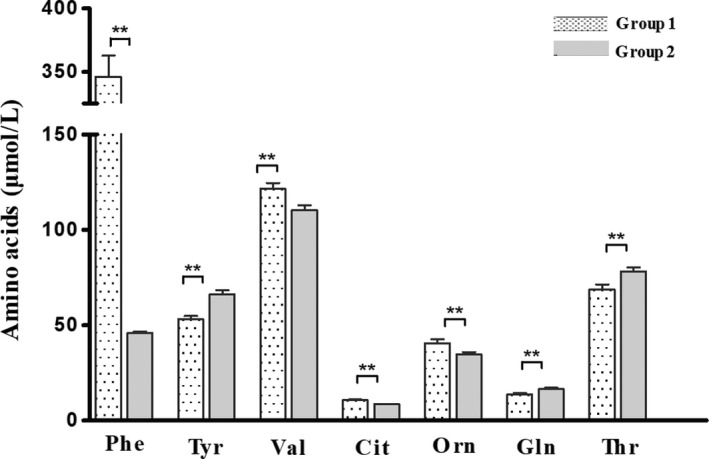
Differences in amino acid levels between HPA newborns and controls. Data are presented as mean ± SEM. **P < .01; *P < .05

The blood amino acid concentrations in the four groups are summarized in Table 2. Although the levels of other amino acids, except for Phe, in newborns were normal, a significant difference in the levels of several amino acids was observed between the patients in this group and those of group 2. The Phe concentration in group 1 (346.30 ± 16.45 µmol/L) was higher than that of group 2 (45.90 ± 0.84 µmol/L), keeping a significant statistical difference (*P* < .0001). Meanwhile, Tyr levels were significantly lower in group 1 than in group 2 (52.97 ± 1.93 vs 66.12 ± 2.19 µmol/L, *P* < .0001). Furthermore, several other amino acids also showed notable difference between HPA newborns and controls. Compared with group 2, group 1 had significantly higher concentrations of Cit, Val, and Orn and significantly lower levels of Gln and Thr (Table 2, Figure 1).

**Table 2:** Amino acid levels in PAH deficiency newborns and controls (groups 1 and 2)

Amino acids	Normal reference for newborn (µmol/L)	Group 1 (Mean ± SEM, µmol/L)	Group 2 (Mean ± SEM, µmol/L)	*P*
Phe	20‐120	346.30 ± 16.45	45.90 ± 0.84	<.0001
Tyr	25‐225	52.97 ± 1.93	66.12 ± 2.19	<.0001
Ala	70‐350	181.60 ± 5.50	178.40 ± 4.10	.6370
Asp	15‐100	37.62 ± 1.52	36.12 ± 0.93	.3799
Glu	100‐500	229.90 ± 6.18	227.50 ± 3.90	.7369
Met	9‐45	21.66 ± 0.62	20.91 ± 0.41	.2971
Leu	55‐300	128.50 ± 3.20	124.50 ± 2.70	.3329
Trp	10‐65	43.04 ± 2.34	41.46 ± 1.21	.5221
Val	40‐250	121.50 ± 3.00	110.30 ± 2.44	.0039
Arg	3‐50	7.50 ± 0.63	6.11 ± 0.45	.0657
Cit	5‐40	10.72 ± 0.43	8.61 ± 0.21	<.0001
Gly	150‐700	314.00 ± 10.23	298.20 ± 5.94	.1615
Orn	20‐160	40.67 ± 1.93	34.68 ± 1.17	.0059
Gln	2‐35	13.60 ± 0.79	16.55 ± 0.68	.0047
His	13‐250	86.06 ± 5.78	95.99 ± 4.45	.1673
Ser	40‐360	127.80 ± 5.57	129.00 ± 3.57	.8462
Thr	25‐160	68.68 ± 2.64	78.21 ± 2.21	.0057

**Figure 2:**
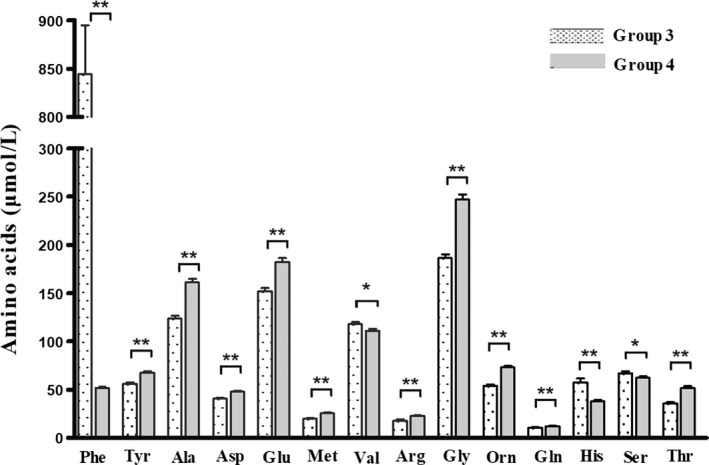
Differences in amino acid levels between older HPA patients and controls. Data are presented as mean ± SEM. **P < .01; *P < .05

The amino acid spectrum of  HPA patients varied with age. The older  HPA (group 3) had different amino acid spectrum compared with HPA newborns. Compared with group 1, Phe concentrations in group 3 increased to 844.00 ± 50.72 µmol/L. The concentration of Tyr in group 3 was 55.97 ± 1.53 µmol/L. The blood concentrations of Phe and Tyr in group 4 were 51.82 ± 1.131 and 67.31 ± 1.82 µmol/L respectively. The difference in Phe concentrations between group 3 and group 4, and the difference in Tyr concentrations between group 3 and group 4 were both significant, despite the fact that the Tyr concentrations in group 3 remained normal. Similar to the newborn groups, although the levels of other amino acids (except Phe) detected in group 3 were normal, several amino acids showed a significant difference from group 4. Compared with group 4, group 3 showed significantly higher levels of His, Val, and Ser and lower levels of Thr, Ala, Asp, Glu, Met, Arg, Gly, Orn, and Gln (Table 3, Figure 2).

**Table 3:** Amino acid levels in older PAH deficiency patients and controls (group 3 and 4)

Amino acids	Normal reference for children (µmol/L)	Group 3 (Mean ± SEM, µmol/L)	Group 4 (Mean ± SEM, µmol/L)	*P*
Phe	20‐120	844.00 ± 50.72	51.82 ± 1.13	<.0001
Tyr	20‐100	55.97 ± 1.53	67.31 ± 1.82	<.0001
Ala	60‐300	123.30 ± 2.87	161.10 ± 3.36	<.0001
Asp	10‐80	40.43 ± 1.11	47.66 ± 1.24	<.0001
Glu	45‐200	151.60 ± 3.62	182.10 ± 4.17	<.0001
Met	8‐35	20.04 ± 0.55	25.59 ± 0.54	<.0001
Leu	50‐250	114.50 ± 2.34	110.30 ± 2.18	.1934
Trp	10‐75	24.20 ± 0.90	24.88 ± 0.50	.4836
Val	80‐300	117.60 ± 2.23	110.90 ± 2.23	.0353
Arg	3‐50	17.94 ± 1.29	22.47 ± 0.85	.0026
Cit	7‐35	17.25 ± 0.53	17.10 ± 0.44	.8288
Gly	90‐350	186.30 ± 3.87	247.00 ± 5.17	<.0001
Orn	15‐80	53.77 ± 1.72	72.93 ± 1.67	<.0001
Gln	6‐30	10.41 ± 0.47	11.91 ± 0.36	.0099
His	10‐300	57.45 ± 3.87	38.10 ± 0.90	<.0001
Ser	20‐100	67.03 ± 1.99	62.36 ± 1.36	.0465
Thr	15‐100	35.94 ± 1.12	51.89 ± 1.54	<.0001

### 3.3 Correlation between Phe and the different amino acid levels in patients

The correlation between Phe and these differential amino acid levels in groups 1 and 3 were estimated using smoothing curve fitting and threshold effect analysis. To determine the linear correlation between the different levels of amino acids and different concentrations of Phe, we calculated the coefficient R and significance level (*P* value) using Pearson's regression. In patients, to determine the nonlinear correlation between the levels of differential amino acids and different concentrations of Phe, we analyzed the threshold effect. The results are summarized in Tables 4, 5, 6.

**Table 4:** Linear correlation between the differential amino acid levels and Phe concentrations in PAH deficiency patients

Amino acids	Group 1		Group 3	
	*R*	*P*	*R*	*P*
Asp			−0.09	.27
Glu			0.04	.61
Val			0.00	.98
Arg			−0.16	.04
Cit	0.11	.26		
Orn			−0.09	.27
Gln	−0.10	.28		
His			−0.11	.16
Ser			−0.13	.10

**Table 5:** Threshold effect of the nonlinear correlation between different amino acid levels and Phe concentration in group 1(*p <.05; **p<.01)

Amino acids	Inflection point (K, µmol/L)	B (95% CI) *P* value (<K)	B (95% CI) *P* value (>K)
Tyr	327.87	−0.10 (−0.16 to −0.04) .0015^**^	0.00 (−0.03 to 0.04) .8599
Val	357.38	−0.03 (−0.11 to 0.05) .4328	0.10 (0.04 to 0.16) .0020^**^
Orn	254.4	−0.11 (−0.21 to −0.01) .0403^*^	0.02 (−0.01 to 0.05) .1873
Thr	152.04	−1.39 (2.44 to −0.34) .0106^*^	−0.01 (−0.04 to 0.02) .6114

**Table 6:** Threshold effect of the nonlinear correlation between different amino acid levels and Phe concentration in group 3 (^*^p <.05; ^**^p<.01)

Amino acids	Inflection point (K, µmol/L)	B (95% CI) *P* value (<K)	B (95% CI) *P* value (>K)
Tyr	748.74	−0.03 (−0.04 to −0.01) .0010^**^	0.01 (−0.00 to 0.01) .0600
Gly	1749.00	−0.02 (−0.04 to −0.00) .0204^*^	0.06 (0.01 to 0.10) .0100^*^
Gln	304.04	−0.02 (−0.04 to −0.00) .0189^*^	−0.00 (−0.00 to −0.00) .0332^*^
Thr	745.80	−0.02 (−0.03 to −0.01) .0025^**^	0.00 (−0.00 to 0.01) .8941

**Figure 3:**
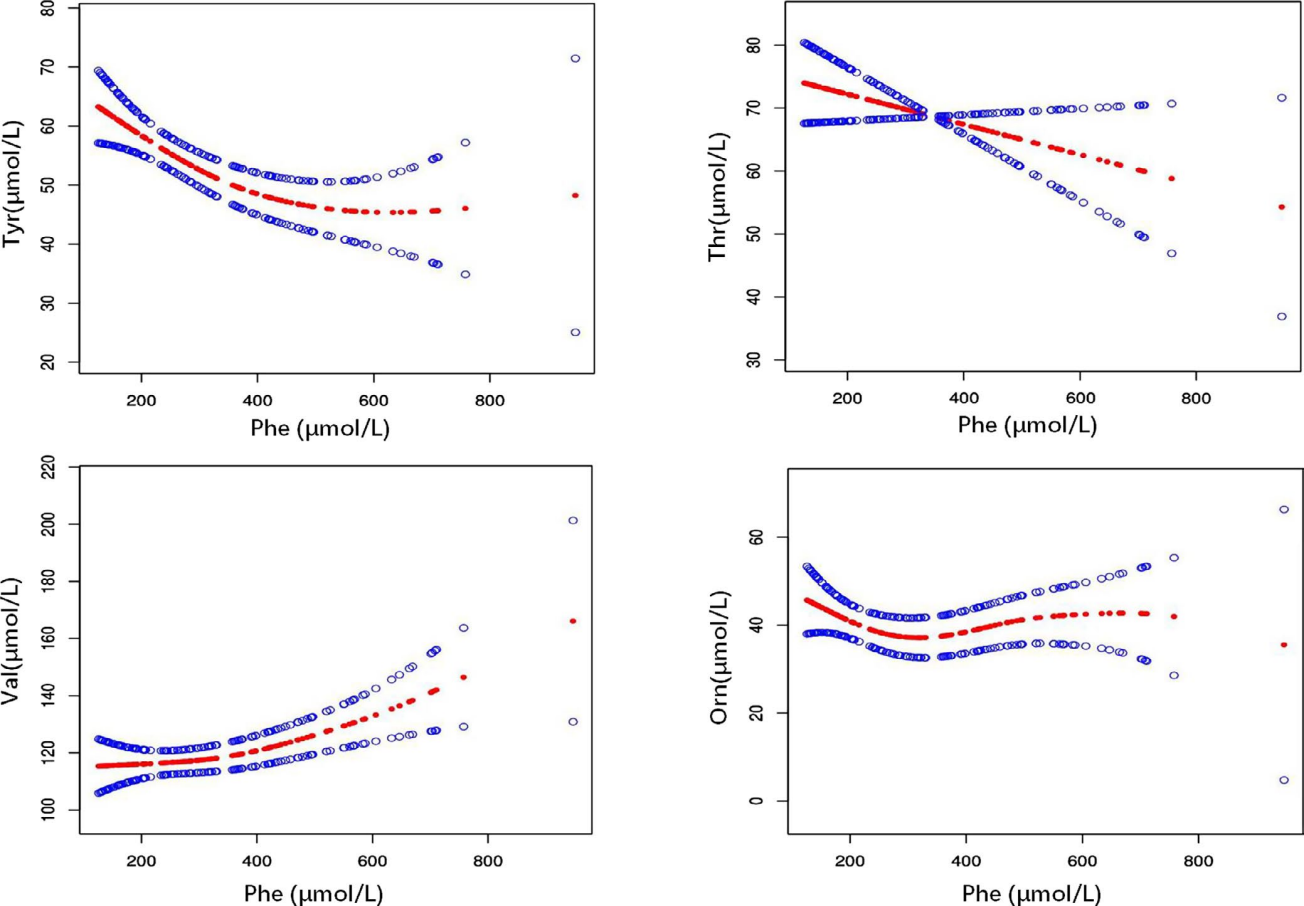
The smooth curve showing the nonlinear correlation between different amino acid levels and Phe in group 1. The four smooth curves represent the correlation between Phe and Tyr, Thr, Val, and Orn, respectively. Red points: smooth curve of amino acid; blue points: 95% confidence interval

Group 1 had higher concentrations of Cit, Val, and Orn and lower levels of Tyr, Gln, and Thr than those of group 2. With regard to these six amino acids in group 1, the concentrations of Cit and Gln showed no linear correlation with the different concentrations of Phe. However, the concentration of Tyr, Val, Orn, and Thr showed obvious threshold effects on the different Phe concentrations. There were different inflection points in the smooth curve of these four amino acids (Figure 3). For example, the smooth curve of Tyr showed an inflection point when the Phe concentration was 327.87 µmol/L. That is to say, when the Phe concentration was <327.87 µmol/L, the Tyr concentration decreased along with an increase in Phe concentration (B = −0.10, 95% confidence interval (CI) = −0.16 to −0.04, *P* = .0015); when the Phe concentration increased further above 327.87 µmol/L, the tendency for Tyr concentration to change disappeared. Similarly, the smooth curve of Thr showed an inflection point when the Phe concentration was 152.04 µmol/L. That is to say, when the Phe concentration was less than this point, the Thr concentration decreased along with an increase in Phe concentration (B = −1.39, 95% CI = −2.44 to −0.34, *P* = .0106). However, when the Phe concentration was higher than the above concentration, the tendency for Thr concentration to decrease along with an increase in Phe concentration also disappeared. The smooth curve of Orn showed an inflection point when the Phe concentration was 254.40 µmol/L. When Phe was <254.40 µmol/L, Orn decreased with an increase in Phe (B = −0.11, 95% CI = −0.21 to −0.01, *P* = .0403). Once the Phe concentration is higher than the above concentration, the said tendency disappeared. In contrast, Val showed an inflection point when the Phe concentration was 357.38 µmol/L in the smooth curve. Val increased along with the increase in Phe concentration, but only when the Phe concentration was >357.38 µmol/L (B = 0.10, 95% CI = 0.04 to 0.16, *P* = .0020).

**Figure 4:**
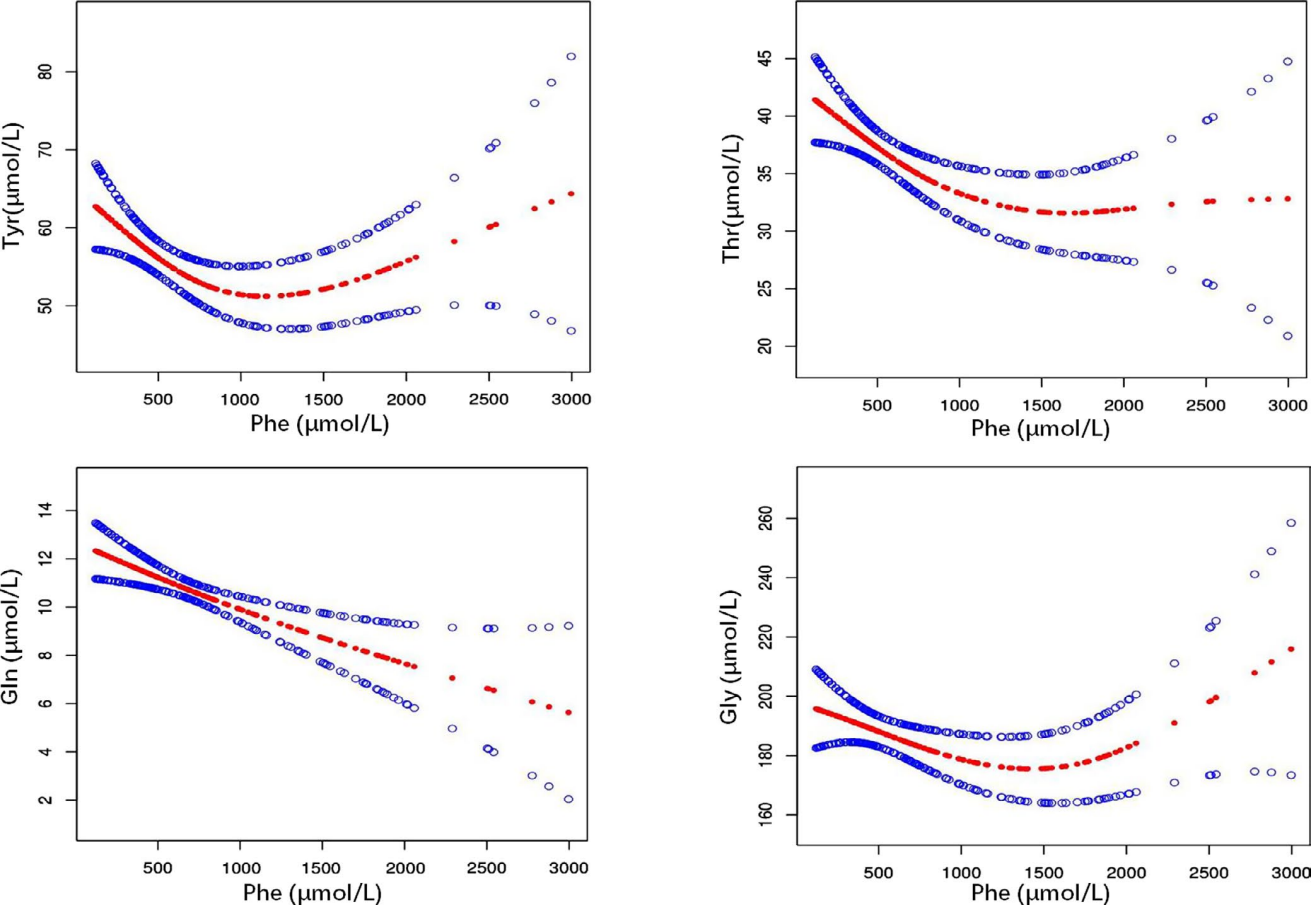
The smooth curve showing the nonlinear correlation between different amino acid levels in group 3. The four smooth curves represent the correlation between Phe and Tyr, Thr, Gln, and Gly, respectively. Red points: smooth curve of amino acid; blue points: 95% confidence interval

Compared with group 4, group 3 had significantly higher levels of His, Val, and Ser and lower levels of Thr, Ala, Asp, Glu, Met, Arg, Gly, Orn, and Gln. We analyzed these differential amino acid levels in group 3 using smoothing curve fitting and threshold effect analysis. Results showed that the concentrations of Asp, Glu, Val, Arg, Orn, His, and Ser had no linear correlation with the concentrations of Phe. However, the concentration of Tyr, Thr, Gly, and Gln had an obvious threshold effect on different Phe concentrations. There were different inflection points in the smooth curve of these four amino acids. As shown in Figure 4, the correlation between Tyr and Phe showed a turning point. As the Phe concentration increased, Tyr decreased and then eventually increased. The rough point of Phe concentration was 748.74 µmol/L. That is to say, when the Phe concentration was <748.74 µmol/L, the Tyr concentration decreased along with the increase in Phe concentration (B = −0.03, 95% CI = −0.04 to −0.01, *P* = .0010). Once the Phe concentration is more than the value, Tyr showed a slight tendency to increase along with the increase in Phe concentration (B = 0.01, 95% CI = 0.00 to 0.01, *P* = .0600). Similarly, the smooth curve of Thr showed an inflection point when the Phe concentration was 745.80 µmol/L. When the Phe concentration was less than this value, the Thr concentration decreased with an increase in Phe concentration (B = −0.02, 95% CI = −0.03 to −0.01, *P* = .0025). When the Phe concentration was more than this concentration, the tendency also disappeared. Moreover, Gln showed an inflection point when the Phe concentration was 304.04 µmol/L in its smooth curve. Gln decreased along with the increase in Phe concentration, but only when the Phe concentration was <304.04 µmol/L (B = −0.02, 95% CI = −0.04 to 0.00, *P* = .0204). Furthermore, the smooth curve of Gly showed an inflection point when the Phe concentration was 1749 µmol/L. When the Phe concentration was <1749 µmol/L, Gly decreased with an increase in Phe concentration (B = −0.02, 95% CI = −0.04 to −0.00, *P* = .0204); when the Phe was more than this concentration, Gly increased along with an increase in Phe concentration (B = 0.06, 95% CI = 0.01 to 0.10, *P* = .0100).

To summarize the general impression of the patients in the two groups, higher Phe was associated with lower levels of Tyr, Thr, Orn, Gly, and Gln in a nonlinear pattern. Moreover, different threshold Phe concentrations existed in their respective smooth curves.

## 4 DISCUSSION

The present study focused on the initial amino acid status of pretherapeutic HPA children, early to several days after birth. Although all the other amino acids determined, except for Phe, were within the normal ranges, many amino acids were significantly different from those of the normal group. In addition to higher Phe and lower Tyr in  HPA patients, HPA newborns (group 1) had higher levels of Cit, Val, and Orn and lower concentrations of Gln and Thr than normal newborns. Meanwhile, older HPA children (group 3) showed higher levels of His, Val, and Ser and lower concentrations of Thr, Ala, Asp, Glu, Met, Arg, Gly, Orn, and Gln than their age‐matched controls. Furthermore, although no linear correlation existed between Phe concentrations and the levels of other amino acids, some amino acids showed notable threshold effect on Phe concentrations in a nonlinear pattern, with clear inflection points. Using the threshold effect analysis, we obtained a general impression that higher Phe concentration was associated with lower Tyr, Thr, Orn, Gly, and Gln levels in a nonlinear pattern. Most of the involved amino acids were LNAA. This finding indicated that Phe produces a pathological effect through LNAA even before treatment.

In a recent study by Suat Ekin on PKU patients, with a mean age of 6.92 ± 0.61 years (n = 30), a tendency of low levels of serum Ala, Gly, and Orn was exposed in patients, although no statistical difference was observed between the PAH deficiency group and healthy group.^21^ In our study, the older  HPA children showed significantly lower levels of Tyr, Ala, Gly, Orn, Thr, Asp, Glu, Met, Arg, and Gln than in the corresponding control group, all with statistical difference. In this point, the results of the two studies were mutually supportive. A statistical difference was observed in our study, while the previous study showed otherwise; we attributed this finding to the sample size used in this study (n = 277), which was larger than that of the previous study (n = 30).

Tyr is synthesized from Phe for normal person. However, the synthesis of Tyr from Phe is impaired in PKU, resulting in lower blood Tyr levels. In our study, Tyr decreased significantly without accident (*P* < .0001). Interestingly, the correlation between Tyr and Phe showed a turning point. Accompanying with the Phe increasing, Tyr concentration first decreased and then eventually increased. The inflection point of Phe concentration was 327.87 µmol/L in group 1 and 748.74 µmol/L in group 3 (Figure 3). We speculated that this might be attributed to the compensatory effect of high Phe on PAH.

Although within normal range, Gln in the blood of both group 1 and group 3 was significantly lower than its respective control groups. Gln is the precursor of Glu, which is the main excitatory neurotransmitter in the human brain.^1^ Just like Thr, Gln is also one of the LNAAs competing with Phe in the process of transporting to the brain; we speculated that the decrease of Gln in blood may lead to its weaker competition with Phe to get into the brain, which can lead to poor glutamate synthesis in the brain. Glu levels in group 1 were within normal range and were similar to that of group 2. However, in older groups, Glu level in group 3 was obviously lower than that in group 4, although the value was still within normal range. The relative lack of Glu might further weaken their competitiveness as neurotransmitters. This finding is similar to that of a previous study and showed that glutamatergic synaptic transmission is impaired in the brain of PKU patients.^1^, ^17^ The relative decrease of Gln and Glu in the blood exposed in our study might be one of the causes of this impairment or may aggravate it.

Arg is a semi‐essential factor because it may not be synthesized sufficiently in growing children.^22^ In our study, Arg level in group 3 was lower than that in group 4, while Arg level in group 1 was similar to that in group 2. These results indicated that more and more nutritional problems gradually developed, along with the continuous accumulation of pathological stimulations. Similar inconsistent variations between different ages occurred in Cit and Orn. The levels of Cit and Orn were higher in group 1 than in group 2, while the levels of Orn were lower in group 3 than in group 4. Although no significant difference was observed in the Arg levels between group 1 and group 2, group 1 exhibited higher levels of Orn, Arg, and Cit (the three amino acids included in the urea cycle) than did group 2. Furthermore, the spectrum varied with age. In older children, although still within normal range, group 3 had lower Arg and Orn levels than those of group 4. As Cit and Orn are important intermediate molecules in the urea cycle, further study is warranted to evaluate whether the urea cycle in HPA children is impaired.

Thr levels in both group 1 and group 3 were significantly lower than the levels of respective control groups. Thr can convert to pyruvate and produce Gly and acetyl‐CoA, which are the most important intermediate molecules in energy metabolism. Thr is an essential amino acid. Decrease in Thr levels might lead to poor protein synthesis and energy metabolism disorder. Since Thr is one of the LNAAs competing with Phe in the process of transporting to the brain, we speculated that a decrease in serum Thr levels may lead to its weaker competition with Phe, and thus only lesser amounts of Thr will be transported into the brain, leading to poor protein synthesis in the brain. However, the mechanism behind the reduction in serum Thr levels of HPA patients remains unclear. In our study, group 3 had lower Gly levels (186.30 ± 3.87 µmol/L) than group 4 (247.00 ± 5.17 µmol/L), with the changes consistent with Thr. This finding is possibly due to the fact that Thr can be converted to Gly. Along with the increase in Phe, both Thr and Gly showed the same trend; that is, they initially decrease and then eventually increase (Figure 3). The consistency of trends observed for Thr and Gly fully confirms this point.

Metabolic syndrome including obesity and insulin resistance is increasing in PKU due to the following factors: diet and the disease itself. Kanufre demonstrated that PKU patients with excess weight showed higher blood concentrations of basal insulin.^23^ Val is associated with insulin resistance: higher levels of Val are observed in the blood of patients with insulin resistance. Kara R. Vogel found that the serum Val levels of PKU mice were elevated.^24^ Similarly, in our study, the levels of Val in group 1 and group 3 were significantly higher than those of their respective control groups. This phenomenon in our study supported the insulin elevation in PKU patients reported in the previous study. We speculated that the elevation of Val might have a correlation with the insulin levels in HPA patients, which requires further study to confirm this result.

Ala is closely related to glucose metabolism. In mammals, Ala plays a key role in glucose–Ala cycle, which is important for gluconeogenesis and energy supply. Besides, pyruvate kinase is the key enzyme in the process of glycolysis. The previous study has indicated that Phe inhibits pyruvate kinase and decreases glycolysis and energy production. Ala, a known competitor of Phe on the enzyme activity, prevents the inhibitory effect of Phe on the enzyme activity and prevents the reduction of glycolysis and energy production caused by Phe.^25^, ^26^ In our study, group 3 had significantly lower Ala levels than controls. We speculated that the decreased level of Ala might lead to a weaker protective function of glycolysis and energy production against Phe for HPA patients. Further study is needed to confirm this finding.

The current study did not include the comprehensive mechanism, but only described the amino acid spectrum in HPA patients before treatment. Overall, several variations were observed in the amino acid spectrum of pretherapeutic  HPA children. Disturbance of amino acids in older patients was more serious than that in newborn patients. Compared with their controls, the relative lack of Thr, Ala, Asp, Glu, Met, Arg, Gly, Orn, and Gln in the blood of patients suggested that the nutritional problems might have occurred before the diagnosis and treatment. These findings might provide new insights into the metabolism of brain injury in HPA.

In conclusion, this study suggests that although the levels of amino acids (except Phe) in pretherapeutic HPA patients remained normal, significant differences existed already in many amino acids, compared with their respective controls. HPA patients before treatment had different amino acid spectrum even at neonatal period. The pretherapeutic amino acid spectrum of patients varied obviously with age. In  HPA neonatal patients, the levels of some amino acids were higher, while those of other amino acids were lower than the amino acid levels in normal newborns. As the patients’ age increased, levels of more amino acids decreased, including Tyr, Thr, Ala, Asp, Glu, Met, Arg, Gly, Orn, and Gln. Some amino acids, especially several LNAAs, showed notable threshold effect with Phe level in a nonlinear pattern. The disturbed pretherapeutic amino acid status might partly be contributed to the occurrence of brain injury in PKU patients. Our findings indicated that more attention should be paid to the nutritional status of HPA patients as early as possible.

## CONFLICT OF INTEREST

The authors report no conflicts of interest.
